# "The Empire of Childhood"

**Published:** 1888-11-03

**Authors:** 


### The Hospital

A Weekly Institutional Journal of

**Science, Medicine, Nursing, and Philanthropy.**

*For Hospitals, Asylums, Medical Practitioners, Students, Nurses, Families, Parishes, Congregations, and the Charitable Public.*

Vol. V. No. 110.] [November 3, 1888.

### "The Empire of Childhood."

By a happy inspiration the Baroness Burdett-Coutts has expressed in a single phrase that infinite variety of facts, experiences, hopes, and fears which centres in and encloses the young. "The Empire of Childhood" gives us as in one complete picture the youthful world within the world of stern manhood: the world that will be within the world that is. It was not, however, with the object of publishing a well-turned phrase that the Baroness wrote to the *Times* a few days ago. Her purpose was to call attention to the case of Louisa Eldridge, and, by means of it, to arouse the thinking and feeling public to the helpless condition of the children of the vicious, and to the state of the law, which is comparatively impotent for their help and protection.

Louisa Eldridge is a little girl ten years old. Most readers of the newspapers will remember that her father died a week or two ago, and that when he was lying dead in the house the mother was drunk. It is not easy to imagine anything much sadder than these circumstances of a child of ten in the same house and the same room, with a dead father, and a mother intoxicated and worse than dead. But the woman herself imagined something unspeakably more dreadful. She conceived the idea of making the child sleep all night in the same bed with the dead man's body. With the determination of drunken madness, she insisted upon her freak being carried out. With such materials, Edgar Allen Poe would have constructed a tale which would have made the flesh creep with horror.

This is a sample of what is going on in London. Let pitying readers for a few minutes give their attention to the "Empire of Childhood." It is a world as well as an empire—a world with two hemispheres. In the one hemisphere it presents a picture of happiness ideal and almost complete. The physical wants are abundantly and intelligently supplied—the need for play, for variety, for boisterous excitement, for entire self-abandonment, is met in a thousand ways. The opening and expanding mental faculties are led on, encouraged and gently stimulated, or checked, in strict accordance with the dictates of a rational physiology, and with the minimum of toilsome and unpleasant effort. In winter, the school with all its modern pleasures, in summer the country and the sea, the fields and the woods, the trout stream and the tennis-lawn, await the happy boy or girl of the prosperous and educated class. It is difficult to think of circumstances better fitted to produce unqualified pleasure than those which environ the young of the well-to-do people of this country. There never was a period in the history of the world when these children were so completely happy as they are at the present time.

That is one picture. Now turn to another. Let it be remembered that all children at birth have the same capacity and the same necessity for wholesome food, careful nursing, baby pleasures, girlhood's joys, boyhood's delights, as the children of the rich. The helpless baby born in a London slum may enter the world with a native intelligence equal to that of a Gordon, a Shaftesbury, or a Florence Nightingale. That pure and innocent new-born mind may be tortured, twisted, battered and destroyed so entirely that the grown man shall die by the hangman, or live to be a Whitechapel murderer. Let it be remembered that children born into certain environments are as powerless to direct their own course, and to escape from their environments as a buoy chained to the bottom of the sea, and tossed in every direction by the resistless might of its never-resting waves. There are hundreds of human parents who have no parallel among the fiercest and least intelligent of wild beasts, and thousands of young children whose lot is such that it would be a merciful kindness to kill them swiftly in cold blood.

Certain persons say it always has been so, and it always will be so. To say that is foolish; to act as if nothing were possible to be done is base and inhuman. The Society for the Prevention of Cruelty to Children has not acted in this *non possumus* and shameful spirit. It is not an old, nor, we believe, a rich Society. But it has a few people within its ranks who are made of worthy and heroic stuff. During the last three years it has "dealt with upwards of 800 cases," says the Baroness Burdett-Coutts, who is one of its members. These cases have varied in degree, though they have been similar in kind to that of Louisa Eldridge. The Baroness has been commissioned by the Society to call public attention to the case of this little girl, in order to claim and secure the help of the public in the passing of a Bill, which the Society has now in hand, through the two Houses of Parliament. This is what the public have to consider: If they begin at once to interest themselves in the Bill, and to urge it upon the sympathy and practical help of their friends, it is quite sure to pass, and that immediately; if, on the other hand, they take no interest in it, and do not make any efforts to awaken interest in their friends, the passing of the Bill may be indefinitely postponed.

We make a direct appeal to our readers. Here is a Society whose objects are of the purest and the most noble kind, and whose *personnel* and accomplished work are such as to command universal sympathy and confidence. The Society is not working for itself or for the benefit, pecuniary or otherwise, of its officials. It is doing the work which the public ought to do. It is charging itself with labours, cares, and responsibilities which rightly belong to the public. It is struggling in stern conflict with enemies of a kind whose baseness, stupidity, inhumanity, and helplessness, are indescribable. The conflict is such, that no man or woman would take part in it unless impelled by motives of a kind which are practically irresistible. The question for us and our readers to consider is this: Shall we leave a little band of heroic men and women to struggle alone with their heart-breaking but generous and noble work, or shall we respond to the appeal of a tried and proved philanthropist, and use all the possible weight at our command to influence the great council of the nation on the children's behalf?

The Autumn Session of Parliament is rapidly approaching. Almost immediately, too, the Local Government Act will come into force. There are thus two instruments lying ready to hand whereby those who really wish and resolve to come at once to the help of the children may forward their designs. But the immediate object to be aimed at is the passing of the Bill which will be brought before the House of Commons within the next few weeks. That object it is every person's interest, as it is everybody's duty, to make sure of. It is no party object. There is only one thing which can prevent the passing of the Bill, and that is public indifference. As everyone knows, Parliament is overworked, and, like every other overworked organisation, it does that thing first which it is most pressed and urged to do. There is no use in waiting for a time of legislative leisure. That time is farther off than even the millennium. The one thing to do is to picture to the mind's eye the sufferings of tens of thousands of helpless and hopeless children, and to hear in imagination their desparing and heart-breaking cries. The passing of the present Bill will be one practical step taken for their deliverance. Let that practical step be made sure beyond possibility of failure by each reader applying to the Baroness Burdett-Coutts, 1, Stratton Street, W., for a petition to Parliament in favour of the Bill. They should next secure as many signatures as possible, and then forward it to the M.P. for the district where they reside.

